# Similar Brain Activation during False Belief Tasks in a Large Sample of Adults with and without Autism

**DOI:** 10.1371/journal.pone.0075468

**Published:** 2013-09-20

**Authors:** Nicholas Dufour, Elizabeth Redcay, Liane Young, Penelope L. Mavros, Joseph M. Moran, Christina Triantafyllou, John D. E. Gabrieli, Rebecca Saxe

**Affiliations:** 1 Department of Brain and Cognitive Sciences, Massachusetts Institute of Technology, Cambridge, Massachusetts, United States of America; 2 Department of Psychology, University of Maryland, College Park, Maryland, United States of America; 3 Department of Psychology, Boston College, Boston, Massachusetts, United States of America; 4 Psychology Department, Harvard University, Cambridge, Massachusetts, United States of America; 5 McGovern Institute for Brain Research, Massachusetts Institute of Technology, Cambridge, Massachusetts, United States of America; University College London, United Kingdom

## Abstract

Reading about another person’s beliefs engages ‘Theory of Mind’ processes and elicits highly reliable brain activation across individuals and experimental paradigms. Using functional magnetic resonance imaging, we examined activation during a story task designed to elicit Theory of Mind processing in a very large sample of neurotypical (N = 462) individuals, and a group of high-functioning individuals with autism spectrum disorders (N = 31), using both region-of-interest and whole-brain analyses. This large sample allowed us to investigate group differences in brain activation to Theory of Mind tasks with unusually high sensitivity. There were no differences between neurotypical participants and those diagnosed with autism spectrum disorder. These results imply that the social cognitive impairments typical of autism spectrum disorder can occur without measurable changes in the size, location or response magnitude of activity during explicit Theory of Mind tasks administered to adults.

## Introduction

Theory of Mind (‘ToM’) is the capacity to represent mental states, such as thoughts, beliefs, desires, feelings, plans, suspicions and doubts [1]. Consideration of others’ mental states helps people in many everyday activities: teaching, flirting, coordinating and cooperating, playing games, conducting minor and massive deceptions, making moral judgments and appreciating fiction. Individuals with autism spectrum disorders (ASD) have impaired ToM. For example, children with ASD are disproportionately delayed on tasks that tap inferences about other people’s beliefs [2]. The neural mechanism of this impairment remains unknown. However, in neurotypical (NT) adults and children, fMRI studies reveal a remarkably reliable group of brain regions recruited during a ToM task of belief reasoning [3]–[9]. These regions include the left and right temporo-parietal junction (RTPJ and LTPJ), right anterior superior temporal sulcus (rSTS), the medial precuneus (PC), and the medial prefrontal cortex (MPFC).

Previous authors have suggested that ToM impairments in ASD could be caused by impaired function in the brain regions typically involved in ToM [10]–[13]. Attempts to characterize the function of these ToM-relevant brain regions in adults with ASD have yielded conflicting results, however. Some studies suggest that activations in ToM regions show no difference between ASD and NT individuals [14], [15]. Others find reduced activity (i.e. hypo-activity) [13], [16], or the opposite pattern, hyper-activity, in ASD [17], [18], while still others find evidence of all three patterns depending on the specific task demands [16], [19].

One factor contributing to these conflicting results may be that sample sizes are small, and individual variability is large. Small samples of individuals with ASD are problematic because individuals with ASD may be highly heterogeneous in their neural responses (e.g., [20]). Small samples of NT individuals are equally problematic, because they allow for calculation of only the mean of the typical response, not its distribution. Understanding the typical distribution is critical if neural measures are to be useful in a clinical or diagnostic setting. For most clinical applications, it is less important to describe differences between groups of individuals (e.g. studies of this nature have an average of 14 adults with ASD vs. 14 NT adults [13]), and more important to be able to describe the neural activity pattern of each specific individual, relative to typical and atypical distributions. For example, using fMRI to help diagnose an individual with ASD would require comparing each individual to the typical distribution.

In the current study, we therefore aggregated data collected over 8 years from 462 NT participants. This large sample allowed us to investigate individual differences in neural responses to a belief-reasoning ToM task, and measure any difference between NT participants and high-functioning adults with ASD with unusually high sensitivity. We also tested whether the response of ToM regions in NT individuals is related to basic demographic factors that may be relevant for ASD, including gender, age, and IQ.

## Methods

All studies whose data are used in the current paper were reviewed and approved by the MIT IRB, the Committee on the Use of Human Experimental Subjects. Participants provided written, informed consent, in accordance with the guidelines of the MIT Committee on the Use of Human Experimental Subjects, and were compensated monetarily for their time.

### Typical Participants

Data were analyzed from 462 NT participants (mean = 24.9 years, range: 18–69 years; 223 male). IQ was measured in 61 of these participants using the Kaufman Brief Intelligence Test (KBIT-2, IQ mean = 117.2, range: 82–134, SD = 12.2). NT Participants are summarized in Table 1.

**Table 1:** Demographic Summary.

Group Name	N	N Male	N ASD	Mean Age	Age Range	Mean IQ	IQ Range	Mean ADOS COMM	Mean ADOS SOC
NT	462	197	0	24.4 (7.4)	18–69				
ASD	31	26	31	32.5 (12.4)	18–66			3.2 (1.3)	5.9 (2.1)
Matched–ASD	27	22	27	31 (11.5)	18–52	117.9 (12.7)	90–141	3.3 (1.2)	5.9 (2.2)
Matched–NT	27	22	0	30.6 (9.3)	18–50	115.1 (12.2)	82–133		
IQ	91	59	30	28.6 (9.9)	18–66	117.1 (13.4)	69–141		

The demographic information for the participant sample and relevant participant subsamples are presented, with standard deviation in parentheses. ADOS COMM = ADOS communication score, ADOS SOC = ADOS social score.

### ASD Participants

31 participants with a clinical diagnosis of ASD (mean = 32.5 years, range: 18–66 years; 26 male) were included in this analysis, having volunteered to participate in one of three previous studies [21]–[23]. In addition to a clinical diagnosis of ASD, the Autism Diagnostic Observation Schedule (ADOS) was administered (ADOS communication score mean = 3.2, SD = 1.3; ADOS social score M = 5.9, SD = 2.1). Each ASD participants had a combined social and communicative score > = 7 (the criterion for inclusion in the study). IQ was measured for all but one male participant with ASD (KBIT-2, IQ mean = 116.8, 69–141, SD = 15.7). In previous studies in our lab, these participants were found to have significant behavioral deficits in ToM [21], [22] in a moral judgment task.

For direct NT vs. ASD comparison, a set of 27 NT participants were chosen based on pairwise match with 27 ASD participants on IQ (±10 points), age (±5 years), and gender. The pairs were also matched on all experimental parameters (e.g. the coil used, the TR and slice thickness, the modality of the stimuli, the number of stimuli per condition, the presentation duration of the stimuli, and the task the participant performed) (Note several of the ASD participants were excluded since they could not be matched to a specific NT participant). These samples each contained 22 males, and were matched on age (ASD mean age = 31.0 years, range 18–66, SD = 11.5; NT mean age = 30.6 years, range 19–50, SD = 9.3) and IQ (ASD mean IQ = 117.9, 90–141, SD = 12.7; NT mean IQ = 115.1, 83–133, SD = 12.2); these 54 participants were termed the ‘matched’ sample.

### fMRI Tasks

All participants were presented with verbal stories in English that described a character who acquired a false belief (Belief condition) or a physical representation that became false, such as an outdated photograph or map (Photo condition). For example, one Belief story was: “The morning of high school dance Sarah placed her high heel shoes under her dress and then went shopping. That afternoon, her sister borrowed the shoes and later put them under Sarah’s bed.” One sample Photo story was: “Sargent famously painted the south bank of the river in 1885. In 1910 a huge dam was built, flooding out the whole river basin, killing the old forests. Now the whole area is under water.” (More example stimuli are available at http://saxelab.mit.edu/superloc.php).

Across conditions, the stories were matched for length (see Table 2 for more details about the tasks). Each participant read or heard an equal number of stories in the two conditions. Localizers were designed to present between 10 and 16 stories per condition to participants, though due to extenuating circumstances a small number of participants were presented with as few as 5 stories per condition while others saw as many as 24 (mean = 13.2). The stories were presented either visually as text on a screen (to 420 participants), or aurally through headphones (to 73 participants). In separate blocks of the same experiment, 121 participants also saw stimuli from other conditions (e.g. physical descriptions of objects, lists of unconnected words) but those conditions were not included in the current analyses. The duration of the stimulus block corresponded, on average, to 0.47 seconds per word (STD = 0.06 s), followed by 10–12 seconds of rest (these values were constant within each variant of the task, see Table 2). After reading or hearing each story, participants performed one of three tasks: true/false (TF, e.g. “In the painting the south bank of the river is wooded. True/False”, N = 304), fill in the blank (FITB, e.g. “In the painting the south bank of the river is… Wooded/Flooded”, N = 101), or word match-to-sample (MTS, e.g. in the preceding story, did you read “Painted”?, N = 88). Task was held constant within participant, but varied across participant. These tasks correspond to the functional localizers used in previously published studies [24]–[30].

**Table 2:** Task Variants.

ID	MeanWords	N StimuliPer Cond	Blocklength (s)	TaskType	Modality	IncludedOther Conds	N Subj	N Male	N ASD	Mean Age(years)	N 32Channel
**A**	36	12	24	FITB	V	Y	33	22	18	33.03	0
**B**	57.9	12	38	MTS	V	Y	88	34	0	24.58	29
**C**	31.2	12	24	FITB	V	N	68	28	0	22.28	0
**D**	30.7	12	28	TF	A	N	65	35	9	30.12	0
**E–A**	30.5	16	28	TF	A	N	8	3	0	22.62	0
**E–V**	30.5	16	24–28	TF	V	N	167	72	1	22.9	0
**F**	31.0	10	24	TF	V	N	64	29	3	24.13	45
**Overall**	35.95	13.16	27.25				493	223	31	24.9	74

The data analyzed here are aggregated across seven variants of the Theory of Mind task. Although the conceptual contrast was constant (Belief>Photo), across participants there was variation in the length and number of stimuli, the modality (V = visual, A = auditory) and explicit task (FITB = fill in the blank, MTS = match to sample, TF = true/false). We also report the mean number of words per stimulus (“Mean Words”), the number of stimuli per condition (“N Stimuli Per Cond”), the Block length, whether or not the experiment included conditions other than false belief and false photo (“Included Other Conds”), and the number of participants scanned with the 32-channel coil on a given localizer (“N 32 Channel”).

### fMRI Methods

Participants were scanned on a 3T Siemens scanner at the Martinos Imaging Center at the McGovern Institute for Brain Research at the Massachusetts Institute of Technology. NT participants were scanned between 2006 and 2013. ASD participants were scanned between 2007 and 2013. Matched NTs were scanned between 2007 and 2013. Functional data were acquired using echo-planar-imaging with voxel size of 3.125×3.125 mm and TE = 30 ms, flip angle = 90°, TR = 2 s. Slice thickness varied from 3.1 mm to 4.8 mm (mean 4.0 mm, SD 0.2 mm) Participants were scanned on either a 12-channel (N = 419) or a 32-channel receive coil (N = 74), both Siemens products. Data were analyzed using SPM8 (http://www.fil.ion.ucl.ac.uk) and in-house software. The data were realigned to account for motion, smoothed with a 5 mm Gaussian smoothing kernel and normalized to a standard template in Montreal Neurological Institute space.

### ROI Analyses

Seven functional ROIs from the ToM network were defined in individual participants, using the contrast Belief>Photo, consistent with previous literature (e.g. [4], [6]): right and left temporoparietal junction (RTPJ, LTPJ), the precuneus (PC), the dorsal, middle and ventral components of MPFC (DMPFC, MMPFC and VMPFC) and the right superior temporal sulcus (RSTS).

To identify individual functional ROIs, initial “hypothesis spaces” for each of the 7 regions were defined based on group random effects analysis and used as a guide to identify clusters of activation representing the ROI in participants. To ensure independence, the participants were split randomly into two groups (first half N = 247, second half N = 246), and the hypothesis space from one group’s random effects analyses was used to define ROIs in the participants belonging to the other group. ASD participants were evenly distributed between the two groups. The hypothesis spaces consisted of all voxels contained in a continuous cluster of suprathreshold voxels that include the region representative of the ROI. The ROI hypothesis spaces were approximately spherical, except the RSTS which was elongated following the sulcus. Averaged across both halves, the DMPFC comprised 1,185 voxels, all z>20 mm, centered at xyz coordinates (−1 mm, 53 mm, 29 mm). The MMPFC comprised 1,094 voxels, between z>0 mm and z<20 mm centered at (1 54 12). VMPFC comprised 774 voxels, all z<0 mm, at (1 50 −12). The RSTS comprised 3,002 voxels, all z>6 mm, centered at (55 −10 −16). The RTPJ comprised 2,812 voxels, all z>6 mm, centered at (54 −52 23). The LTPJ similarly compromised 2,444 voxels, all z>6 mm centered at (−52 −58 25). Finally, the PC hypothesis space consisted of 3,339 voxels centered at (1–56 34). Average ROI hypothesis spaces are available as binary images in the NIfTI-1 file format at saxelab.mit.edu/hypothesis_spaces.zip.

Each participant’s contrast image (Belief>Photo) was masked iteratively with the six hypothesis spaces. After each masking, candidate voxels were identified within the hypothesis space–where a voxel was a candidate if it was individually significant at *p*<0.001 (uncorrected) and contiguous with at least 10 other voxels significant at *p*<0.001. From this set of candidates, the voxel with the peak T is selected, along with all other candidate voxels that are contiguous with and not more than 9 mm from the peak. From each ROI, five parameters were extracted: the size of the ROI (number of voxels included), the mean T value across voxels included in the ROI, and the x-, y-, and z-coordinate of the ROI’s “center of mass,” being the average position of ROI voxels weighted by their T values. The presence or absence of an identified ROI in each region was itself used as an additional parameter.

The reliability of ROI parameters within participants was assessed by split-half analysis. Two contrast images were defined, one from even run data and another from odd run data, in each participant. The correlation of the ROI even and odd parameter values was measured across participants. Significance was established by iteratively permuting (5000 permutations) the even-half data across participants to generate an empirical ‘null’ distribution. We report individual differences as reliable if the true pairing showed a higher correlation than 90% of the empirical null distribution. (Note that since these analyses are based on half of the data per subject, they are conservative estimates of the reliability of individual differences measured based on the full dataset per individual).

Next, we sought (i) to remove variance from the ROI parameters associated with ‘nuisance’ demographic and experimental variables to better reveal differences (if any) between ASD and NT groups and (ii) to evaluate the effects (if any) of our demographic and experimental variables on ROI parameters. To both these ends, a multivariate Generalized Linear Model (GLM) was constructed for each ROI parameter with a nine-column (age, gender, group, modality, coil, number of stimuli per condition, mean words per stimulus, task type and the intercept term) predictor matrix using data from 493 (462 NT and 31 ASD) participants (see Table 3). For the binary statistic that indicated whether or not the ROI of interest was identified in a given subject, the GLM presumed a binomial distribution and a logit linker function. The GLM used a normal distribution and an identity linker function for all other ROI statistics. Regressors, except the intercept, were mean-centered prior to regression. Correction for multiple comparisons was performed with Bonferroni correction for the nuisance predictors, across all predictors (age, gender, etc.) and all dependent measures (mean T, number of voxels, etc.), within each ROI, as detailed below. With the exception of the beta values that relate the predictors to the probability of discovery (which is binomial), these beta values directly relate the size of the effect in parameter units (i.e., mm) per regressor unit (i.e., years). Given the number of predictors being used, we evaluated the estimability of the predictors of interest, particularly the group predictor, using Belsley’s Collinearity test [31]. The group predictor of interest was never found to exceed the standard tolerance (a variance decomposition proportion greater than 0.5 and a condition index greater than 30) established in the MATLAB collintest function. All predictor/parameter pairs were found to lie well within tolerance, across all tests.

**Table 3:** Representation of the analysis methods used in this study.

Sample	N	Analysis Strategy	Predictors Used										
			Group	IQ	ADOS Comm	ADOSSoc	Age	Coil	Gender	Modality	# Stim	# Words	TaskType
**Full**	**NT = 462** **ASD = 31**	**ROI analysis,** **Whole brain**	***X***				X	X	X	X	X	X	X
**Matched**	**NT = 27** **ASD = 27**	**ROI analysis,** **Whole brain**	***X***	X			X		X	X	X	X	X
**IQ**	**NT = 60** **ASD = 30**	**ROI analysis**	X	***X***			X		X	X	X	X	X
**ASD**	**ASD = 31**	**ROI analysis**			***X***	***X***	X		X	X	X	X	X

This table indicates, for each sample reported in the study, the number of participants from each group included (N), which set of analyses were conducted, and the predictors used to account for the observed variance (indicated with an ‘X’). The predictors of primary interest, for each analysis, are marked in bold and italicized; the remaining predictors are treated as exploratory.

In addition to the large-sample GLM, three other samples were considered in turn using an identical procedure, the only variation being the subset of participants from which the predictor matrix was constructed (see Table 1, Table 3). These groups were: (1) the ‘Matched’ sample: 27 ASD participants were matched pairwise to 27 NT participants based on gender, age, IQ, coil, stimulus modality, and task (both ASD and NT participants are drawn from [21]–[23]), (2) the ‘ASD only’ sample: an analysis of variability within participants diagnosed with ASD and (3) the ‘IQ’ sample: including all 91 participants for whom IQ was collected. In each sample, all non-degenerate predictors were used (i.e., predictors whose values were defined in all participants and varied within-group, see Table 3). Estimability was assessed in the same way as in the Full sample. Across all tests, only one predictor, for one parameter, was found to exceed this tolerance: IQ when used to predict the mean T value of the MMPFC in the IQ sample. Thus, our predictors of interest were properly estimable in our models.

Because of the very large number of comparisons, we corrected *p* values using three different correction factors (m) according to Bonferroni’s formula, [corrected *p*] = 1-(1-[uncorrected *p*])^m^. For our key a priori predictors of interest (ASD vs NT in the full sample and the ‘Matched’ sample, and IQ in the ‘IQ’ sample), we corrected for the 6 dependent variables (i.e. the ROI parameters) per ROI, resulting in m = 6. The effect of ADOS score was measured in the ASD-only sample, and since it has two parameters (a social and a communication score), we used m = 12. All of the remaining predictors were treated as exploratory, so effects of these predictors are reported as significant corrected for both the number of dependent variables (6) and the number of nuisance predictors (9), resulting in m = 54 (exploratory predictors are only considered in the full sample). Any relationship found to be significant at *p*<0.01 uncorrected is discussed as a ‘trend,’ though corrected *p*-values are always reported for consistency.

In the matched sample, the significance effect of group on the mean value of each ROI parameter was also measured and multiple-comparison corrected nonparametrically. The objective of such nonparametric tests was to select an alpha using an empirical distribution such that the probability of *any* parameter within an ROI being a false positive result was 0.05. To this end, we permuted the group labeling randomly 25,000 times. In each permutation, the significance of the difference in means between the randomly generated groups was measured by a t-test. This yielded 25,000 *p* values for each parameter within an ROI. The *p* values for that ROI were pooled together and sorted, and the 0.83%tile (i.e., the 5^th^ %tile divided by 6, the number of comparisons per ROI) *p* value was chosen. This *p* value represents an empirical threshold such that, for a given ROI, the chances of obtaining at least one *p* value less than it for any parameter is 5%. We also tested whether the groups differed in the variance of any ROI parameter using a similar strategy, with two important differences: the *p* value was calculated based on an Ansari-Bradley test, a nonparametric two-sample test of equal variances, and the “found/not found” parameter was omitted, since the mean and variance of a vector of 1′s and 0′s are directly related by a deterministic function.

### Whole-brain analyses

Whole brain analyses were conducted on the main contrast of interest (Belief>Photo). To correct for multiple comparisons, nonparametric whole-brain analysis was performed using SnPM (http://www.sph.umich.edu/ni-stat/SnPM/), which estimates the false-positive rate directly from the data. Each test used 3 mm variance smoothing and 5,000 permutations, with no global normalization, grand mean scaling, or threshold masking. The corrected *p*-value for filtering was 0.05, with an uncorrected T-value minimum threshold of 3, and a voxel-cluster combining theta value of 0.5. Voxel-cluster combining was performed jointly by Fisher, Tippet and Mass voxel-cluster combining functions. Permutations were repeated for each predictor of interest; all demographic and experimental predictor variables were included in each model as nuisance regressors using a modified SnPM plugin designed to support nuisance regressors (see Table 3).

To look for subtle group differences, we also conducted a second, more sensitive whole-brain analysis. We used a more lenient voxel-wise threshold (p<0.001 uncorrected) to correct for multiple comparisons, and then validated the results using a split-half analysis. We used data from each participant’s even and odd runs, separately, to identify clusters showing a group difference (NT>ASD, or ASD>NT) in the response to Belief>Photo stories. We identified clusters in either the even or odd run random effects analyses, and extracted the response in those clusters in the other half of the data; clusters are reported as significant if the corresponding group difference was observed in the left out data at p<0.05 uncorrected.

## Results

### ROI results

Six functional ROIs (ROIs) from the ToM network were defined in individual participants, using the contrast Belief>Photo, consistent with previous literature [4], [6]: RTPJ (in 464/493 individual participants, or 94%), LTPJ (87%), PC (91%), DMPFC (68%), MMPFC (64%), VMPFC (55%) and RSTS (85%). In the matched sample, ROI definition was successful in both groups: RTPJ (NT: 96%, ASD: 96%), LTPJ (89%, 82%), PC (82%, 93%), DMPFC (74%, 70%), MMPFC (48%, 59%), RSTS (93%, 74%).

The goal of this project was to explain individual differences in the size, magnitude and/or position of brain regions involved in ToM. Before testing individual differences, however, it was critical to determine that (i) there was variability in these measures, and (ii) the differences between participants on these measures are reliable (i.e. that inter-individual differences do not simply reflect noise in the measurement). All ROI parameters showed reasonable variability. The standard deviation of the mean T-value ranged between 0.5 and 1 across ROIs, and the standard deviation of ROI size (measured in number of voxels) ranged between 60 and 100 voxels. In order to test whether this variability reflects stable individual differences, we compared the correlation of ROI parameters from independent halves of the data from the each individual to an empirical permutation-based ‘null’ distribution of these correlations. Both mean T and ROI size were reliable within individual, compared to variability across participants, for all ROIs (mean T: all r>0.25, rank>96%; size: all r>0.13, rank>90%), except VMPFC. Center of mass was somewhat less reliable: the x position was reliable (rank>90%) for RTPJ, LTPJ and MMPFC; the y position was reliable for RTPJ, PC, DMPFC, MMPFC, and RSTS; and the z position was reliable for RTPJ, DMPFC, VMPFC, and RSTS.

Next we used multivariate GLM analyses to estimate whether any variance in the size, position or response magnitude of ToM brain regions is explained by whether an individual has been diagnosed with ASD.

**Figure 1:**
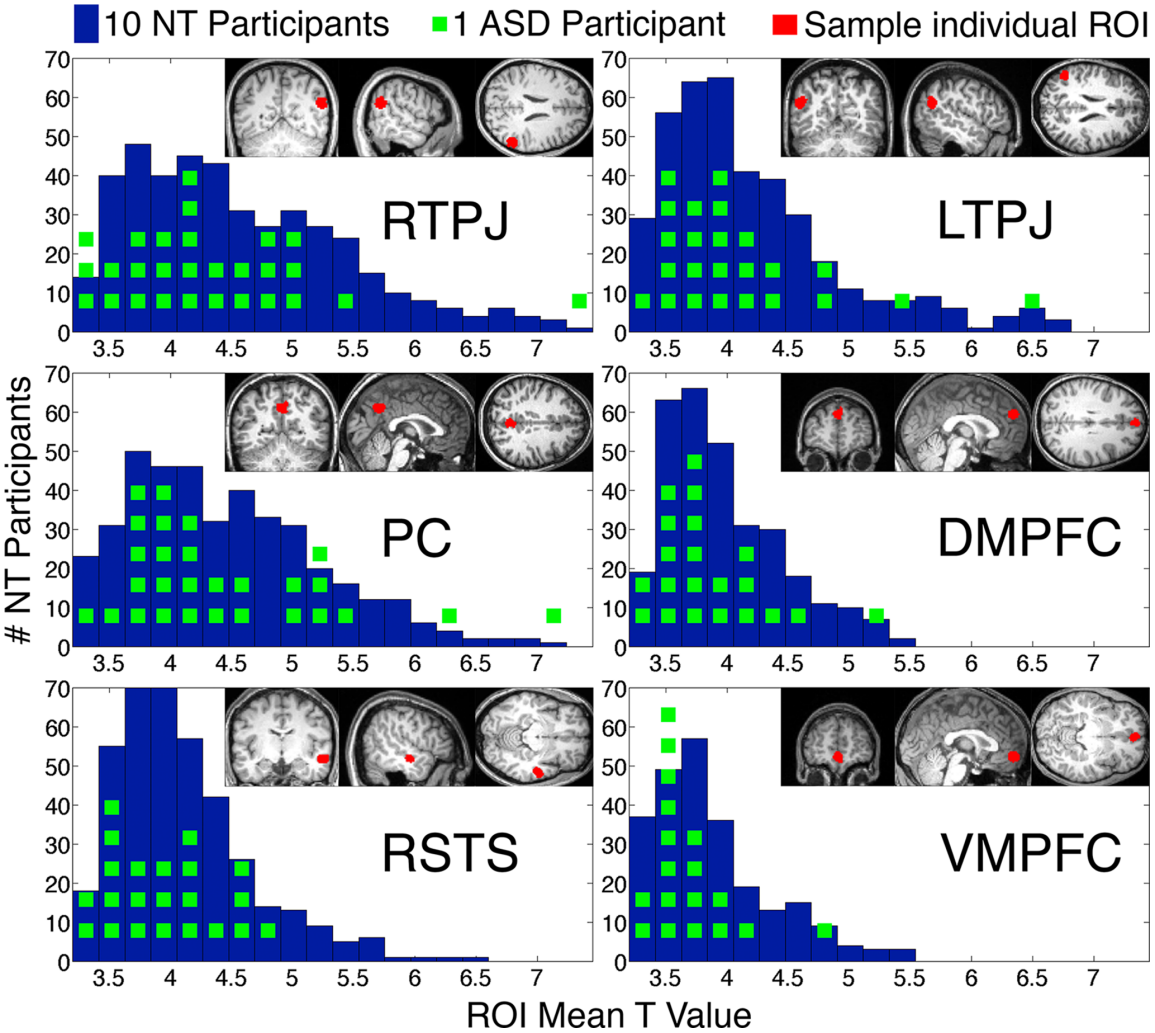
Histograms of ROI mean T-values for six Theory-of-Mind ROIs individually defined in each participant. The T-values are from the Belief>Photo contrast in each participant. The minimum T-value for defining an ROI was 3. Blue bars show NT participants while green squares show individual ASD participants. Insets show individually defined ROI for a representative participant. The regions shown are: (top left) right temporoparietal junction, (top right) left temporoparietal junction, (middle left) precuneus, (middle right) dorsal medial prefrontal cortex, (bottom left) ventral medial prefrontal cortex, and (bottom right) right anterior superior temporal sulcus.

For the large sample analysis, we compared all of the participants with ASD (N = 31, 26 male) to all of the NT participants (N = 462, 197 male). No parameter of any ROI was significantly predicted by the group membership (ASD vs. NT) of the individual (all *p*>0.22 for all ROIs, see Figure 1, Table 4). Furthermore, the odds ratio favoring the null hypothesis (no difference between the distributions) over the alternative hypothesis (a difference between NT and ASD), for all regions and all parameters was greater than 1.8∶1 (Bayes factor, [32]), with two exceptions: for the mean T in VMPFC (0.8∶1) and the probability of finding RSTS (1.1∶1) the odds of the null and alternative hypotheses were approximately equal. No ASD participant fell outside of 3 standard deviations of the typical distribution on any measure for any ROI. The confidence intervals on the coefficient estimates were quite small, indicating a high degree of confidence that if any differences exist, those differences are very small. For instance, if there exists a difference in the mean T value of the RTPJ voxels between ASD and NT participants, we are 99% certain this difference is less than a T value of.3 in either direction.

**Table 4:** Summary of effects of group membership on ROI parameters in the Full and Matched sample.

Full Sample										
ROI	Parameter	Beta	T value	P value	Lower CL	Upper CL	Degrees Freedom	# NT	# ASD	Baye's Factor
RTPJ	Mean T	0.101	1.030	0.885	−0.153	0.356	455	436	28	4.048
	# Voxels	17.160	1.679	0.447	−9.276	43.595	455	436	28	1.778
	X Center	0.463	0.757	0.972	−1.120	2.047	455	436	28	5.090
	Y Center	0.207	0.316	1.000	−1.488	1.903	455	436	28	6.353
	Z Center	−1.001	−1.317	0.715	−2.967	0.965	455	436	28	2.955
	Found	0.300	0.818	0.960	−0.649	1.249	484	462	31	5.089
LTPJ	Mean T	0.073	0.723	0.978	−0.189	0.335	422	407	24	4.872
	# Voxels	12.161	1.272	0.746	−12.575	36.897	422	407	24	2.930
	X Center	1.091	1.494	0.584	−0.799	2.980	422	407	24	2.204
	Y Center	−0.128	−0.191	1.000	−1.867	1.611	422	407	24	6.107
	Z Center	0.621	0.813	0.961	−1.355	2.596	422	407	24	4.569
	Found	0.298	1.118	0.841	−0.391	0.987	484	462	31	3.872
PC	Mean T	0.025	0.255	1.000	−0.226	0.276	437	418	28	6.450
	# Voxels	2.874	0.270	1.000	−24.654	30.401	437	418	28	6.426
	X Center	0.128	0.255	1.000	−1.175	1.432	437	418	28	6.450
	Y Center	−0.523	−0.852	0.951	−2.109	1.064	437	418	28	4.731
	Z Center	0.965	1.053	0.875	−1.407	3.338	437	418	28	3.956
	Found	−0.065	−0.188	1.000	−0.956	0.826	484	462	31	6.859
DMPFC	Mean T	0.097	1.371	0.675	−0.086	0.281	328	316	21	2.453
	# Voxels	6.730	0.871	0.945	−13.291	26.752	328	316	21	4.106
	X Center	0.116	0.126	1.000	−2.277	2.509	328	316	21	5.779
	Y Center	0.096	0.167	1.000	−1.394	1.586	328	316	21	5.747
	Z Center	−0.982	−1.430	0.633	−2.762	0.797	328	316	21	2.272
	Found	−0.092	−0.418	0.999	−0.658	0.475	484	462	31	6.423
MMPFC	Mean T	0.120	1.509	0.572	−0.086	0.327	308	299	18	1.931
	# Voxels	13.463	1.504	0.578	−9.745	36.672	308	299	18	1.945
	X Center	−0.140	−0.172	1.000	−2.255	1.975	308	299	18	5.368
	Y Center	−0.112	−0.170	1.000	−1.830	1.605	308	299	18	5.369
	Z Center	0.117	0.175	1.000	−1.612	1.845	308	299	18	5.365
	Found	0.084	0.398	0.999	−0.460	0.627	484	462	31	6.473
VMPFC	Mean T	0.148	2.056	0.222	−0.039	0.335	260	251	18	0.794
	# Voxels	13.533	1.643	0.476	−7.840	34.905	260	251	18	1.588
	X Center	−0.308	−0.707	0.980	−1.440	0.823	260	251	18	4.313
	Y Center	−0.056	−0.085	1.000	−1.749	1.638	260	251	18	5.396
	Z Center	−0.651	−0.959	0.916	−2.414	1.111	260	251	18	3.564
	Found	−0.032	−0.157	1.000	−0.566	0.502	484	462	31	6.894
RSTS	Mean T	0.064	0.851	0.951	−0.130	0.257	410	397	22	4.277
	# Voxels	7.437	0.941	0.922	−13.009	27.883	410	397	22	3.969
	X Center	−0.535	−0.688	0.983	−2.548	1.478	410	397	22	4.802
	Y Center	2.894	1.433	0.631	−2.333	8.121	410	397	22	2.316
	Z Center	−1.609	−1.217	0.782	−5.030	1.812	410	397	22	3.014
	Found	0.476	1.977	0.256	−0.147	1.099	484	462	31	1.108
**Matched Sample**										
**ROI**	**Parameter**	**Beta**	**T value**	**P value**	**Lower CL**	**Upper CL**	**Degrees Freedom**	**# NT**	**# ASD**	**Baye's Factor**
RTPJ	Mean T	0.098	0.842	0.955	−0.215	0.412	44	26	26	3.511
	# Voxels	2.261	0.194	1.000	−29.165	33.687	44	26	26	4.746
	X Center	0.335	0.348	1.000	−2.257	2.927	44	26	26	4.570
	Y Center	0.878	0.917	0.934	−1.700	3.456	44	26	26	3.310
	Z Center	−0.906	−0.939	0.927	−3.502	1.691	44	26	26	3.251
	Found	−0.282	−0.346	1.000	−2.469	1.906	46	27	27	4.650
LTPJ	Mean T	0.036	0.312	1.000	−0.281	0.354	38	24	22	4.375
	# Voxels	−2.482	−0.232	1.000	−31.513	26.549	38	24	22	4.461
	X Center	1.391	1.318	0.728	−1.470	4.252	38	24	22	2.129
	Y Center	0.328	0.349	1.000	−2.219	2.875	38	24	22	4.327
	Z Center	−0.754	−0.723	0.979	−3.585	2.077	38	24	22	3.623
	Found	0.220	0.498	0.997	−0.968	1.409	46	27	27	4.387
PC	Mean T	0.073	0.500	0.997	−0.322	0.469	39	22	25	4.121
	# Voxels	−1.212	−0.080	1.000	−42.268	39.843	39	22	25	4.595
	X Center	0.942	1.351	0.707	−0.946	2.831	39	22	25	2.064
	Y Center	−0.198	−0.238	1.000	−2.459	2.062	39	22	25	4.493
	Z Center	0.293	0.201	1.000	−3.646	4.232	39	22	25	4.525
	Found	−1.348	−2.000	0.246	−3.159	0.463	46	27	27	0.849
DMPFC	Mean T	0.113	1.194	0.809	−0.146	0.372	31	20	19	2.294
	# Voxels	14.279	1.022	0.896	−24.041	52.598	31	20	19	2.701
	X Center	0.346	0.341	1.000	−2.439	3.131	31	20	19	4.044
	Y Center	−0.086	−0.107	1.000	−2.280	2.108	31	20	19	4.235
	Z Center	−0.994	−1.065	0.877	−3.554	1.566	31	20	19	1.949
	Found	−0.002	−0.005	1.000	−0.900	0.897	46	27	27	4.908
MMPFC	Mean T	0.141	1.685	0.493	−0.096	0.378	21	13	16	1.171
	# Voxels	14.182	1.201	0.812	−19.258	47.621	21	13	16	2.049
	X Center	0.641	0.483	0.998	−3.120	4.403	21	13	16	3.389
	Y Center	0.067	0.068	1.000	−2.736	2.871	21	13	16	3.735
	Z Center	−0.549	−0.487	0.997	−3.738	2.641	21	13	16	3.383
	Found	−0.180	−0.617	0.990	−0.964	0.604	46	27	27	4.134
VMPFC	Mean T	0.145	1.659	0.500	−0.097	0.386	27	19	16	1.272
	# Voxels	14.575	1.530	0.590	−11.818	40.967	27	19	16	1.506
	X Center	−1.045	−1.712	0.461	−2.737	0.647	27	19	16	1.184
	Y Center	−0.392	−0.361	1.000	−3.396	2.613	27	19	16	3.830
	Z Center	−0.994	−1.029	0.894	−3.671	1.682	27	19	16	2.571
	Found	0.241	0.733	0.976	−0.641	1.122	46	27	27	3.851
RSTS	Mean T	0.036	0.513	0.997	−0.155	0.227	37	25	20	4.007
	# Voxels	0.590	0.063	1.000	−24.973	26.154	37	25	20	4.497
	X Center	0.182	0.165	1.000	−2.818	3.182	37	25	20	4.451
	Y Center	1.453	0.530	0.996	−5.985	8.890	37	25	20	3.976
	Z Center	−0.564	−0.311	1.000	−5.493	4.365	37	25	20	4.316
	Found	0.740	1.556	0.536	−0.538	2.017	46	27	27	1.675

For each ROI/parameter pair, the effect of group membership was measured via a GLM. The estimated beta value, the t− and p- values, the 99% confidence intervals, and the number of NT and ASD individuals included in the regression are reported. Also reported is the Baye’s factor, which relates the odds ratio of the null hypothesis (group membership has no effect) to the alternate hypothesis.

Next we compared participants with ASD to NT participants in the ‘Matched’ group. Again, we found no significant difference between participants with ASD and the matched controls on any ROI parameter (all *p*>0.24, Table 4). For these comparisons, the odds ratio favored the null hypothesis over the alternative hypothesis (i.e. ratio>1.1∶1) for all regions and all parameters, with one exception, the probability of finding activity in the PC (0.85∶1). We also confirmed these results using nonparametric tests of group differences (which do not assume that the measured variables are normally distributed and used the null distributions to establish a corrected alpha). No ROI parameter showed an effect of group (the closest to significance was *p*>0.07, against a corrected threshold of *p*<0.01). We conducted a similar analysis to test whether the ASD group showed a more heterogeneous response (i.e. some participants showing hypo-activation while others showed hyper-activation). There was no evidence of increased variance in the ASD group, for any parameter for any ROI (the closest to significance was *p>*0.01, against a corrected threshold of *p*<0.002).

In the ‘ASD only’ group, we next considered the effect of ADOS scores (i.e. social and communicative symptom severity). No significant effects of ASD severity were found. At the level of a trend (i.e. *p*<0.01 uncorrected), an individual’s social ADOS score predicted the position of the RSTS along the STS (along the anterior-posterior axis), with greater social ADOS scores predicting more anterior RSTS ROIs (*t*(14) = 3.03, *p* = 0.11, β = 4.97±4.88 mm/ADOS point).

Finally, the effect of IQ was assessed in the ‘IQ group’. Higher IQ significantly increased the chances of identifying the PC (*t*(83) = 2.76, *p* = 0.03, β = 0.10±0.09), the MMPFC (*t*(83) = 2.88, *p = *0.02, β = 0.06±0.05), and the VMPFC (*t*(83) = 2.74, *p* = 0.04, β = 0.06±0.06). Further, higher IQ predicted significantly greater mean T-value (*t*(74) = 3.35, *p* = 0.008, β = 0.03±0.02) and size (*t*(74) = 3.14, *p* = 0.01, β = 2.63±2.21) of the PC.

As an exploratory analysis, we looked for effects of other demographic and experimental parameters. We found that gender and age did not affect any ROI parameter, even at the level of a trend, nor did the modality of the stimuli.

In the full sample, the variable with the greatest effect was the choice of coil. The 32-channel coil produced ROIs with significantly greater mean T-value in all ROIs except VMPFC (RTPJ: *t*(455) = 7.54, *p*<0.0001, β = 0.57±0.20, LTPJ: *t*(422) = 6.55, *p*<0.0001, β = 0.49±0.19, PC: *t*(437) = 6.05, *p*<0.0001, β = 0. 46±0.19, DMPFC: *t*(328) = 3.92, *p = *0.006, β = 0.21±0.14, MMPFC: *t*(308) = 4.50, *p*<0.0005, β = 0.26±0.15, RSTS: *t*(410) = 4.25, *p*<0.0001, β = 0.36±0.14). RTPJ and RSTS were also significantly larger in participants scanned with the 32-channel coil (RTPJ: *t*(455) = 4.27, *p = *0.0001, β = 33.68±20.42, RSTS: *t*(410) = 4.25, *p = *0.0016, β = 24.85±15.13). All ROIs were numerically, but not significantly, more likely to be found using the 32-channel coil versus the 12-channel coil. Coil choice did not affect the center of mass of the ROIs.

A larger number of words per stimulus slightly but significantly decreased the mean T-value of the RTPJ (*t*(455) = −3.99, *p = *0.004, β = −0.020±0.013). At the level of a trend (i.e. *p*<0.01 uncorrected), a similar effect was observed in the LTPJ (mean T-value: *t*(422) = −3.32, *p*<0.06, β = −0.017±0.013; and size: *t*(422) =  −2.72, *p = *0.3, β = −1.30±1.23). However, the results pertaining to the mean number of words per stimulus come with a caveat. One version of the ToM task (localizer B, see Table 1) had substantially more words per stimulus than any other version (58 vs 31); Localizer B was also the only version that used a Match-to-Sample task, so these effects may be related to stimulus length, task, or the specific stimuli used in this experiment.

The number of stimuli per condition did not predict any ROI parameter significantly. At the level of a trend, the VMPFC tended to have fewer voxels as the number of stimuli increased (*t*(260) =  −2.62, *p = *0.4, β = −4.13±4.10).

In sum, ROI analyses suggest that while individuals differ reliably in the size and response magnitude (and to a lesser extent, position) of brain regions associated with ToM, these neural parameters were not affected by whether an individual was diagnosed with ASD. Within the range of ADOS scores in the current sample, autism severity did not explain variance in these ROI parameters, either. Only experimental parameters, such as the MRI coil used, and demographic variables, such as IQ, explained some of the variance across participants. However, ROI analyses inevitably provide a limited window on the brain, so to look further for differences between groups in ToM brain regions, we conducted whole brain analyses.

### Whole brain analysis results

**Figure 2:**
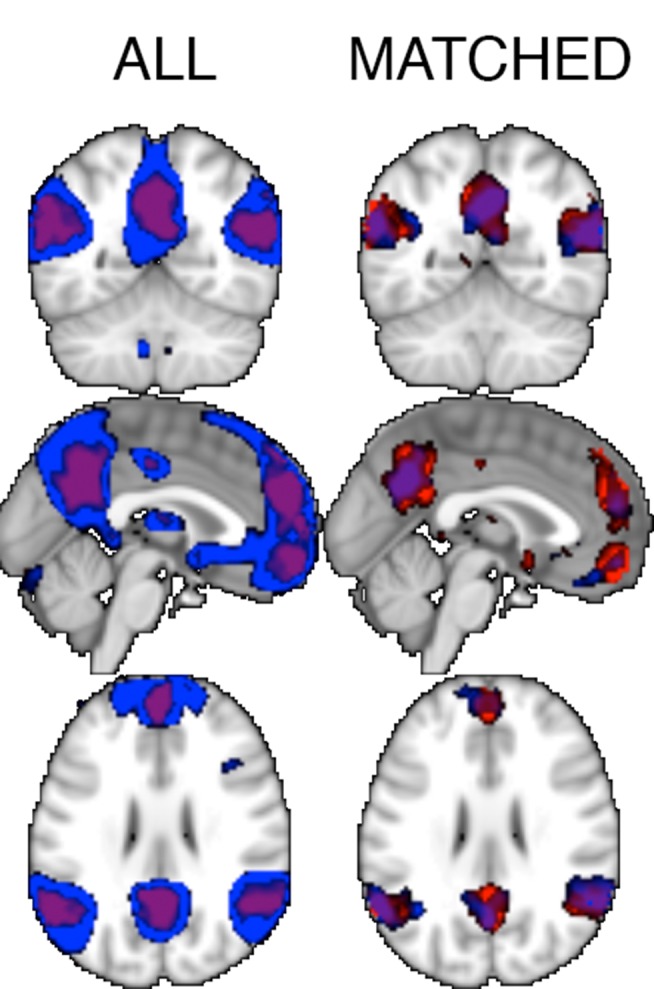
Results of the random effects analysis of the main effect (Belief>Photo). In blue, regions identified in NT participants (N = 462) as responding to the false belief condition more than the false photo condition (*p*<0.001 uncorrected). Results from ASD participants are in red (*p*<0.001, shown in purple because of overlap). Data are overlayed on the MNI template brain. The volume is centered at [0 mm −54 mm 28 mm], showing the LTPJ and RTPJ (visible in the coronal and axial slices), the PC (in all slices) and the MPFC (in the sagittal slice).

In the whole-brain analyses, the main effect identifies brain regions significantly recruited during Belief compared to Photo stories, controlling for variance explained by any of the nuisance regressors. This analysis identified robust activation in all of the regions previously associated with Theory of Mind, including bilateral TPJ, medial precuneus and posterior cingulate, MPFC, and STS (see Figure 2, Table 5). It also identified activation in other regions, including (bilaterally) the hippocampus, the parahippocampal gyrus, the temporal poles, the amygdala, and the dorsolateral prefrontal cortex.

**Table 5:** Table of clusters and peaks identified in the random effects analysis of the main effect, in the matched sample, separated by group and sorted by size.

ASD MATCHED					
X (mm)	Y (mm)	Z (mm)	T-value	# Vox	Region
4	−54	40	9.11	1445	PC
0	−54	34	7.989		
2	−50	28	7.582		
−56	−56	26	7.76	983	LTPJ
−54	−66	32	7.459		
−56	−64	24	7.019		
58	−58	16	8.229	935	RTPJ
62	−52	20	7.142		
54	−52	24	6.049		
2	50	22	9.528	570	DMPFC, MMPFC
0	44	48	5.268		
−12	58	30	4.478		
4	58	−14	7.662	455	VMPFC
2	58	−8	7.485		
−6	54	−14	4.878		
60	−28	−6	6.936	414	RSTS
54	−22	−8	6.06		
50	−22	−10	6.043		
−62	−20	−8	7.279	411	LSTS
−64	−30	−4	5.974		
−56	−30	−6	5.153		
50	12	−36	5.675	115	Temporal Pole
48	20	−32	5.153		
58	6	−20	5.135		
−2	6	−12	5.013	41	Caudate Nucleus
34	26	−20	5.386	30	Right Medial Orbital Gyrus
32	22	−18	5.069		
**NT MATCHED**					
**X (mm)**	**Y (mm)**	**Z (mm)**	**T-value**	**# Vox**	**Region**
60	−6	−12	8.172	2903	RTPJ, RSTS, Temporal Pole
58	−54	16	8.171		
64	−24	−10	8.158		
2	−56	36	6.371	969	PC
0	−60	30	6.34		
−6	−52	46	4.641		
−58	−48	18	7.269	855	LTPJ
−56	−58	24	6.709		
−40	−58	28	5.426		
−62	−16	−4	7.162	660	LSTS
−52	−12	−16	5.683		
−56	−2	−10	5.367		
−6	54	22	5.951	633	DMPFC, MMPFC
6	46	46	5.498		
6	52	20	5.399		
4	52	−12	5.595	157	VMPFC
2	44	−18	5.305		
2	32	−22	4.269		
54	26	−2	6.901	62	Right Insula
42	30	−18	4.823	36	R. Med. Orbital Gyrus

The top three peaks are reported for all clusters identified, where a cluster is defined as a group of at least 30 contiguous suprathreshold (i.e., *p*<0.001, u.c.) voxels. ‘Region’ indicates what region(s) were included in that particular cluster (in some cases, large clusters at this threshold included multiple regions that could be distinguished in individuals).

**Figure 3:**
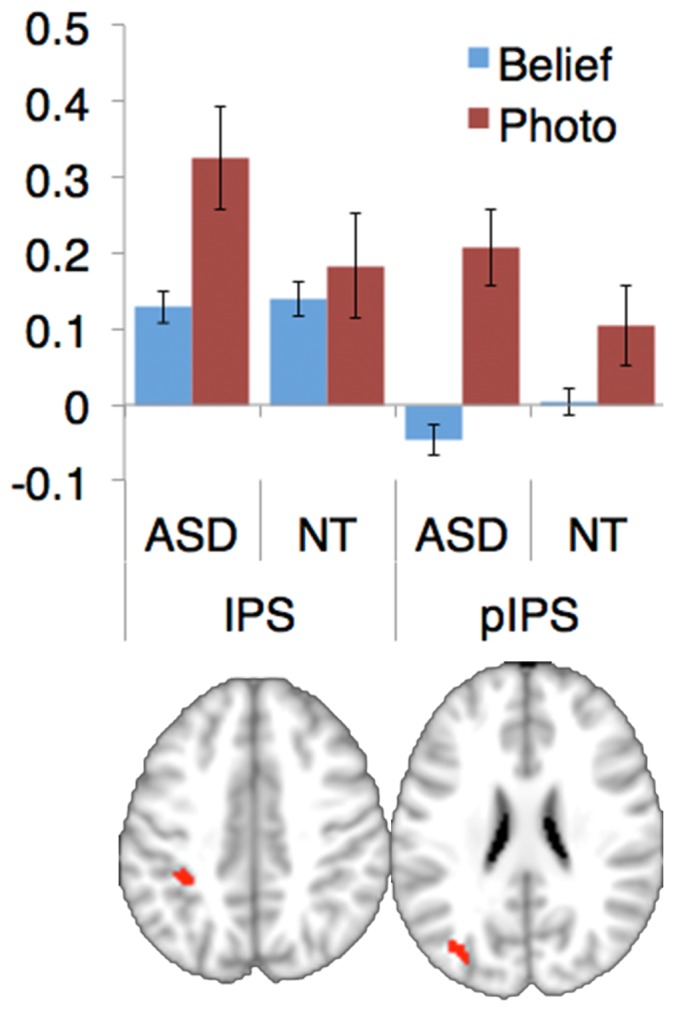
Clusters identified in a split-half analysis for which group has a significant effect on the condition difference. Two clusters, both in the intraparietal sulcus (IPS) were found to be reliable in the split-half analysis. The mean beta, averaged across individuals, for each condition in each group is shown. pIPS denotes posterior IPS.

Next, we compared activation in individuals with ASD vs NT adults, in both the Matched and Full sample. There were no significant differences in activation, when correcting for multiple comparisons. We then repeated the whole brain analysis in the Full sample using a lenient threshold (p<0.001 voxel-wise, uncorrected) in half of the data, and validated the results in the remaining half (p<0.05). Two clusters were identified by the contrast NT>ASD × Belief>Photo: one in left anterior IPS (14 voxels, peak at [−32 mm, −40 mm, 40 mm]), and the other in left posterior IPS (38 voxels, peak at [−34 mm −38 mm 42 mm]). The anterior IPS cluster was identified in both odd and even halves of the data (independent validation in even half: t(462,31) = 3.15, *p* = 0.002), whereas the posterior IPS cluster was found only in the odd half, but validated in the even half (t(462,31) = 2.16, *p = *0.03; see Figure 3). In both regions, both groups showed higher responses to the Photo than the Belief stories, but ASD participant’s greater activation during the Photo stories than NT participants. No regions were reliably recruited more in ASD than in NT individuals, for Belief>Photo.

A variety of other experimental covariates yielded clusters of activation, although we treat these as exploratory. These are listed in Table 6.

**Table 6:** Other clusters identified (Corrected). X, Y, and Z coordinates are mm, in MNI Space. A (−) indicates clusters that correlate negatively with the covariate.

Contrast	X (mm)	Y (mm)	Z (mm)	Nvox	Peak T
Age	−5	−23	20	1111	5.58
	−44	−2	4	533	4.39
Age (−)	0	−57	47	296	4.44
12 ch >32 ch	43	43	21	379	6.23
	38	50	−6	223	4.90
	−42	37	23	214	4.36
	−58	−43	−18	168	4.92
	60	10	9	117	5.10
	34	−48	65	97	4.69
32 ch >12 ch	0	57	16	1821	19.64
	−50	−68	24	340	4.58
	57	4	−20	303	4.68
	52	30	2	302	4.57
	7	61	−22	16	4.95
Male>Female	−41	32	−19	115	4.70
# Words/Stim	−39	42	15	1455	6.62
	−54	−39	45	614	6.61
	−1	39	13	486	5.14
	−51	−54	−12	171	4.94
	45	39	10	158	4.84
# Words/Stim (−)	−2	−80	11	3923	5.84
	51	8	−30	487	5.18
	−52	6	−29	444	5.34
	−58	−7	−20	332	4.60
	57	−5	−20	319	5.75
	−58	−13	−9	251	4.94
	−50	−67	38	173	4.90
	−14	−54	7	163	4.72
	30	−88	−39	65	4.65
	−15	57	38	30	12.64
# Stim	0	−25	28	202	4.58
# Stim (−)	−56	−54	10	264	4.64
	47	−74	28	228	4.79
Auditory>Visual	60	−7	−1	335	5.09
Visual>Auditory	−20	−79	21	378	4.18

## Discussion

The main question we sought to address in this paper was whether adults diagnosed with ASD show differences in the magnitude or location of activations in ToM-associated brain regions, compared to a large sample of NT participants. In order to answer this question, we aggregated data across multiple experiments to produce a large sample of NT individuals (N = 462) and a moderately large sample of high functioning individuals with ASD (N = 31). We tested whether the magnitude of neural responses to stories about people’s beliefs, versus about physical representations like photographs, differed between groups either in targeted regions of interest or in whole brain analyses. These analyses identified no reliable differences between groups in the previously identified ToM brain regions. These results suggest that differences in activation between these groups of participants during explicit Theory of Mind tasks, if they exist, are very small and could not be used to diagnose ASD.

### Effects of ASD on ToM activations

We used two complementary analysis strategies: ROI analyses focused on previous identified ToM brain regions are more sensitive, whereas whole brain analyses look for differences between groups anywhere in the brain, and therefore are less restricted. For both kinds of analyses, we conducted two comparisons. First, we compared the ASD group to the whole group of NT individuals, using simultaneous nuisance regressors to control for variance associated with demographic and experimental differences among participants. Second, we compared the ASD group to a smaller sample of NT individuals, one-to-one matched to the ASD group on age, gender, IQ and experimental parameters. For both comparisons, we found no reliable differences between groups in the size, response magnitude, or probability of identifying above-threshold voxels, in any ToM ROI (see Figure 1).

In addition to the absence of mean differences between the groups, we found no evidence that even a subset of individuals with ASD differed significantly from the typical population. The ROI parameters of individuals with ASD fell squarely within the distribution of typical values, rarely straying more than 2 SD from the typical means and never more than 3 SD. We also tested the hypothesis that the ASD group was more heterogeneous than the NT group. For example, similar mean activation could mask differences between the groups if the ASD group included a bimodal distribution: some individuals showing hypo-activation while other show hyper-activation. We found no evidence for this hypothesis, as the variance of the ROI parameters did not differ significantly across groups in the matched sample.

In the whole brain analyses, permutation-based correction revealed no significant differences between ASD and NT individuals, in either the full sample or the matched sample. Because our results overall suggest a null result–namely, no difference between groups–we also examined the same analyses at a more lenient threshold in half of the data (in case true differences between groups that are just below the threshold for significance), and then validated in the left-out half. We found two regions of parietal cortex with reliable effects; however, the group differences in these regions were in an unpredicted direction. In both regions, both groups showed more activation during Photo than Belief stories, but the ASD group showed more activation than the NT group during Photo stories. Furthermore, these regions were not near any of the regions implicated in ToM by the overall contrast of Belief>Photo. While intriguing, differences in these regions therefore do not seem likely to explain impairments in ToM typically observed in ASD. We could not identify any region that both (a) was reliably recruited for Belief more than Photo stories in 462 NT individuals, and (b) showed significantly less, or more, activation in the same contrast in 31 individuals with ASD.

### Effects of other experimental parameters on ToM activations

Using a similar analysis strategy, we also found that gender does not affect activity in ToM brain regions; nor do the modality of the stimuli (visual vs. aural) or the experimental task. The absence of an effect of gender is particularly noteworthy, because the full sample contained a large number of male and female participants. Behavioral measures of ToM often reveal an advantage for female individuals [33], [34]; apparently this advantage is not due to measurable differences in ToM-associated brain region activity as elicited by the false belief task.

One factor that did have a significant effect on ROI parameters was the coil used. The 32-channel coil has documented higher SNR [35]; we found that this difference translated into larger ROIs that were more likely to be detected in individual participants. Thus, our results suggest that for individually-defined ROI analyses, the increased SNR of the 32-channel coil provides a clear benefit.

### Interpreting the current results

With regard to our key null results, the current study has advantages and disadvantages. On the one hand, the large sample size provides more power and sensitivity to detect effects where they exist. In particular, although our sample of ASD individuals was only moderately large, the very large sample of NT individuals included gives us very high confidence on the true mean of the ROI parameters in NT individuals. Finding that the ASD population mean does not differ from the NT mean is thus strong evidence that these data cannot be attributed to different population distributions.

However, these results cannot be interpreted as ruling out any differences in the neural mechanisms for ToM in individuals with ASD. One qualification of the current results is that the parameters measured here (i.e. response magnitude to Belief vs. Photo stories) provide only a limited measure of a region’s function. Other measures include the functional connectivity of each region and within-region spatial pattern of responses [36], [37]. Individuals with ASD may differ in these other measures of ToM region function [14], [38]. Indeed using multi-voxel pattern analysis, we found reliable differences between a subset of these same ASD and NT individuals in the pattern of activity in ToM regions [21].

A second qualification is that these results apply to a specific functional task: an explicit, verbal false belief task. It may be that deficits in theory of mind in individuals with ASD disproportionately affect implicit or spontaneous consideration of others’ mental states, but not performance on explicit tasks [39]. fMRI studies using tasks that elicit spontaneous or implicit social processing may be more likely to find hypo-activation [23], [40]–[42], whereas those with tasks that demand explicit social judgments find normal or hyper-activation [19]. For example, spontaneous processing of irony may produce hypo-activation in ASD [43], whereas explicit instructions eliminate the hypo-activation, and may even cause hyper-activation [19].

Finally, a third qualification is that the ASD participants in the current sample are very high functioning. Although they meet clinical diagnostic criteria for ASD (and have been shown to have behavioral deficits in ToM tasks in a previous study [22]), these individuals are highly verbal and pass first-order false belief tasks. Thus, our results do not rule out gross differences in the ToM regions of lower-functioning individuals with ASD. On the other hand, the individuals in our sample are diagnosed with ASD because of disproportionate difficulties with social interaction and communication. Also, we found no evidence that within our participants increasing ASD severity had any effect on the measured ROI parameters. So the current results imply that social cognitive impairments can occur without measurable changes in the magnitude or position of ToM brain regions. Collectively, the current results provide strong evidence that the neural differences between high functioning adults with ASD and NT participants are not due to gross changes in ToM brain regions.

A common hypothesis is that the lack of performance differences between NT and high-functioning ASD individuals is a function of the development of compensatory processes in the ASD individuals. Our findings provide evidence against this hypothesis. Compensation predicts that successful performance on explicit ToM tasks would be supported by activity in other regions than (or in addition to) ToM regions. For example, one possible prediction might be that individuals with ASD pass false belief tasks by recruiting the mechanisms that NT individuals used to solve the logically similar ‘False Photograph’ tasks, such as the fronto-parietal network [44], [45]. By contrast to these predictions, we found no sign of any increased compensatory activation during Belief stories, in ASD compared to NT individuals, in any region.

These results leave open a number of key questions. First, it will be important to identify the neural differences between adults with ASD and NT individuals that do account for behavioral differences in ToM. One possibility is that individuals with ASD are highly heterogeneous, so that different neural sources explain the behavioral delays in different individuals. As noted above, though, we do not see evidence for this possibility in the current data. Another possibility, also discussed above, is that the difficulties in theory of mind processing are related to the online use of these regions in real-world social interactions. It will be important to determine what social contexts lead to atypical as well as typical recruitment of these brain regions in ASD. Perhaps ToM brain regions can be recruited during explicit tasks but atypical interaction with other brain regions and networks results in hypoactivation during implicit tasks. Third, the current study focused on adults. It will be important in future research to test whether the developmental trajectory of ToM brain regions differs in children with ASD compared to NT children, even if the mature states of the system are reasonably similar. Finally, it would be useful to extend these analyses to lower-functioning individuals with ASD.

Nevertheless, the implications of this study are that (i) social-cognitive impairments can occur without large differences in the activation of ToM brain regions; and (ii) hypo-activation during explicit Theory of Mind tasks will not be useful for diagnosing ASD.
